# Aberrant static and dynamic functional patterns of frontoparietal control network in antipsychotic‐naïve first‐episode psychosis subjects

**DOI:** 10.1002/hbm.24992

**Published:** 2020-05-06

**Authors:** Frederic Briend, William P. Armstrong, Nina V. Kraguljac, Shella D. Keilhloz, Adrienne C. Lahti

**Affiliations:** ^1^ Department of Psychiatry and Behavioral Neurobiology University of Alabama at Birmingham Birmingham Alabama USA; ^2^ Department of Biomedical Engineering Georgia Tech and Emory University Atlanta Georgia USA

**Keywords:** antipsychotic‐naïve functional connectivity, dynamic functional connectivity, first‐episode psychosis, quasiperiodic patterns

## Abstract

Psychotic disorders are disabling clinical syndromes characterized by widespread alterations in cortical information processing. Disruption of frontoparietal network (FPN) connectivity has emerged as a common footprint across the psychosis spectrum. Our goal was to characterize the static and dynamic resting‐state functional connectivity (FC) of the FPN in antipsychotic‐naïve first‐episode psychosis (FEP) subjects. We compared the static FC of the FPN in 40 FEP and 40 healthy control (HC) subjects, matched on age, sex, and socioeconomic status. To study the dynamic FC, we measured quasiperiodic patterns (QPPs) that consist of infraslow spatioemporal patterns embedded in the blood oxygen level‐dependent signal that repeats over time, exhibiting alternation of high and low activity. Relative to HC, we found functional hypoconnectivity between the right middle frontal gyrus and the right middle temporal gyrus, as well as the left inferior temporal gyrus and the left inferior parietal gyrus in FEP (*p* < .05, false discovery rate corrected). The correlation of the QPP with all functional scans was significantly stronger for FEP compared to HC, suggesting a greater impact of the QPPs to intrinsic brain activity in psychotic population. Regressing the QPP from the functional scans erased all significant group differences in static FC, suggesting that abnormal connectivity in FEP could result from altered QPP. Our study supports that alterations of cortical information processing are not a function of psychotic chronicity or antipsychotic medication exposure and may be regarded as trait specific. In addition, static connectivity abnormality may be partly related to altered brain network temporal dynamics.

## Graphical abstract

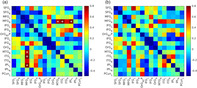

## 1 INTRODUCTION

Traditionally, large‐scale functional brain connectivity has been evaluated by using resting‐state functional magnetic resonance imaging (rs‐fMRI), which assesses the temporal covariation of low‐frequency fluctuations (0.008–0.08 Hz) in the blood oxygen level‐dependent (BOLD) signal across the brain (Biswal, Yetkin, Haughton, & Hyde, 1995). Using this technique, disruption of frontoparietal network (FPN) connectivity has emerged as a common footprint across the psychosis spectrum (Baker et al., 2014; Cole, Repovš, & Anticevic, 2014; Kaiser, Andrews‐Hanna, Wager, & Pizzagalli, 2015; Zeng et al., 2018), with graded disruptions of FPN associated with different forms of psychiatric illness (Baker et al., 2019).

These studies that employed static FC analyses assume constant connectivity patterns over the length of the scan, thus disregarding the dynamic nature of brain activity (Calhoun, Miller, Pearlson, & Adalı, 2014; Hutchison et al., 2013). To address this concern, several studies have now used dynamic functional connectivity (FC) by calculating transient patterns of FC through windowed time course sampling. Clustering these patterns results in connectivity states that are believed to be representative of discrete mental states of connectivity that subjects pass through during the scan (Allen et al., 2014; Calhoun et al., 2014; Hudson, Calderon, Pfaff, & Proekt, 2014; Hutchison et al., 2013). Altered dynamic connectivity has been reported in schizophrenia (SZ) (Du et al., 2018; Lottman et al., 2017; Rabany et al., 2019; Rashid, Damaraju, Pearlson, & Calhoun, 2014), and results suggest that dynamic FC is characterized by weaker connectivity than in healthy controls (HCs) and by shorter mean dwell times.

On the other hand, quasiperiodic patterns (QPPs) consist of infraslow (<0.1 Hz) spatiotemporal patterns embedded in the BOLD signal that repeats over time, exhibiting alternation of high and low activity. QPPs are reproducible patterns of spatial changes (Majeed et al., 2011), distinct from physiological noise and global signal (Yousefi, Shin, Schumacher, & Keilholz, 2018), and ubiquitous across species (mice, Belloy et al., 2018; rats, Majeed et al., 2011; as well as in resting‐state and task‐performing humans, Abbas, Belloy, et al., 2019; Majeed et al., 2011). Because infraslow activity is one of the best candidates for explaining the coordination between large brain networks (Thompson, Pan, Magnuson, Jaeger, & Keilholz, 2014), QPPs could offer a window into the relationship between functional networks. Most importantly, QPPs have been shown to contribute to FC (Abbas, Belloy, et al., 2019). Thus, this technique has the potential to reveal important contributors to resting state functional alteration seen in psychosis.

Here we investigate the resting‐state connectivity and QPP patterns within regions of the FPN in antipsychotic‐naïve first‐episode psychosis (FEP) and matched HCs. The FPN is the portion of the control system involved in highly adaptive processes of goal‐directed task demands (Cole et al., 2013). Its spans portions of the dorsolateral prefrontal cortex, dorsomedial prefrontal cortex, lateral parietal cortex, and posterior temporal cortex. Because the FPN is critical for cognitive performance, and cognitive impairments precede the onset of psychosis in SZ, alteration in this network could shed light into the mechanism underlying the transition to psychosis (Khandaker, Zimbron, Dalman, Lewis, & Jones, 2012; Roiser et al., 2013). Because chronicity and medication status affect the resting‐state FC (Kraguljac et al., 2016, for a summary of studies examining large scale network abnormalities at rest), evaluating antipsychotic‐naïve FEP subjects is especially important in demonstrating that FC constitutes a potential trait alteration in antipsychotic‐naïve FEP. Based on the existing literature (Baker et al., 2014, 2019), we hypothesized that we would replicate findings of reduced connectivity within the FPN in FEP compared to HC. In addition, we hypothesized that we would observe group differences in QPPs, and these differences would explain some of the alterations seen in static FC. Finally, we also conducted exploratory analyses to investigate whether static and dynamic connectivities are associated with clinical variables.

## 2 MATERIAL AND METHODS

### 2.1 Participants

Forty antipsychotic‐naïve FEP patients were recruited from the emergency room, inpatient units, and outpatient clinics at the University of Alabama at Birmingham (UAB). Patient diagnoses were established using diagnostic and statistical manual of mental disorders‐5 criteria by review of medical records, and consensus of two board certified psychiatrists (A. C. L. and N. V. K.). HCs matched on age, gender, and parental socioeconomic status (SES) were recruited by advertisements (Table 1). Exclusion criteria included major neurological or medical conditions, a history of head trauma with loss of consciousness, substance use disorders (excluding nicotine and cannabis) within 6 months of imaging, pregnancy or breastfeeding, or MRI contraindications. HCs with a family history of a psychiatric illness in a first‐degree relative were also excluded. The UAB Institutional Review Board gave approval for this study and written informed consent was obtained prior to enrollment and after subjects were deemed to have capacity to provide consent.

**Table 1:** Demographic and clinical information of the sample

	HC	FEP	*p* Value
	*n* = 40	*n* = 40	
Age (years)	24.77 (6.44)	23.40 (5.80)	*t* test: 0.31
Gender (% males)	62.5	67.5	*χ* ^2^: 0.81
Handedness (% right handed)	100	90	*χ* ^2^: 0.15
SES	4.37 (4.25)	6.52 (5.20)	*t* test: 0.052
Duration of untreated psychosis (weeks)	–	76.55 (172.1)	–
BPRS			
Total	–	52.15 (11.77)	–
Positive	–	11.80 (3.31)	–
Negative	–	6.30 (3.53)	–
RBANS			
Total	95.37 (11.33)	72.08 (11.33)	*t* test: <0.01*
Attention subscale	104.03 (17.58)	77.38 (17.58)	*t* test: <0.01*

Abbreviations: BPRS, brief psychiatric rating scale; FEP, first‐episode psychosis; HCs, healthy controls; RBANS, repeatable battery for the assessment of neuropsychological status; SES, socioeconomic status.

*Note:* Parental socioeconomic ranks were determined from the diagnostic interview for genetic studies (1–18 scale); smaller rank (lower numerical value) corresponds to higher socioeconomic status.

The brief psychiatric rating scale (BPRS) was used to assess symptom severity (Overall & Gorham, 1962). Cognitive function was characterized using the repeatable battery for the assessment of neuropsychological status (RBANS; Randolph, Tierney, Mohr, & Chase, 1998) (Table 1).

### 2.2 Data acquisition and preprocessing

All imaging was performed on a 3T whole‐body Siemens MAGNETOM Prisma MRI scanner. High‐resolution anatomical T1‐weighted magnetization‐prepared rapid acquisition gradient‐echo structural scans were acquired for anatomical reference and morphological analyses (repetition time (TR) = 2,400 ms; echo time (TE) = 2.22 ms; inversion time = 1,000 ms; flip angle = 8°; voxel size = 0.8 mm isotropic; and 256 × 256 matrix). A high‐resolution T2‐weighted image were also obtained (TR = 3,200 ms; TE = 563.0 ms; flip angle = 8°; voxel size = 0.8 mm isotropic; and 256 × 256 matrix). Two fMRI scans were acquired using a gradient recalled echo‐planar imaging sequence (phase‐encoding directions: *A* > *P* and *P* > *A* to correct for magnetic field inhomogeneity; TR = 1,550 ms; TE = 37.80 ms; flip angle = 71°; fields of view = 104 mm^2^; multiband acceleration factor = 4; slice thickness = 2 mm; 225 volumes; and 72 axial slices). During each rs‐fMRI scan, subjects were instructed to keep their eyes open and oriented to a fixation cross.

All preprocessing was conducted using FSL 5.0.9 (Jenkinson, Beckmann, Behrens, Woolrich, & Smith, 2012) and MATLAB according to the automated preprocessing pipeline used in a previous study (Abbas, Bassil, & Keilholz, 2019). Anatomical data were registered to the MNI 152 structural template using FLIRT, skull‐stripped using BET, and tissue segmented into white matter (WM), gray matter, and cerebrospinal fluid (CSF) using FAST. Moreover, functional scans were slice time corrected using FSL's slicetimer tool, motion corrected using MCFLIRT, and spatially smoothed with a 6‐mm Gaussian kernel using FSLMATHS. Band‐pass filtering between 0.01 and 0.08 Hz was applied via MATLAB. Finally, global signal, WM, and CSF were regressed out from the BOLD signal.

### 2.3 Region definition and FC comparison between groups

To create a standard set of regions of interests (ROIs), we use the Human Brainnetome Atlas, a validated connectivity‐based parcellation atlas composed of 210 cortical and 36 subcortical brain regions (Fan et al., 2016). The average time series were extracted for each participant in each region (Jenkinson et al., 2012).

The FPN spans portions of the dorsolateral prefrontal cortex, dorsomedial prefrontal cortex, lateral parietal cortex, and posterior temporal cortex. We defined a set of 16 cortical regions corresponding to the FPN by using a common partition from the Yeo atlas (Yeo et al., 2011). To this end, the 246 ROIs from the Human Brainnetome atlas were consolidated into 16 ROIs based on the structural hierarchy of the atlas. For instance, of the 7 ROIs comprising the middle frontal gyrus (MFG) in each hemisphere (14 total), 3 ROIs were combined into one for the left MFG, and 6 were combined into one for the right hemisphere (all the overlapping is publicly available for download in the Brainnetome Atlas site).

**Figure 1:**
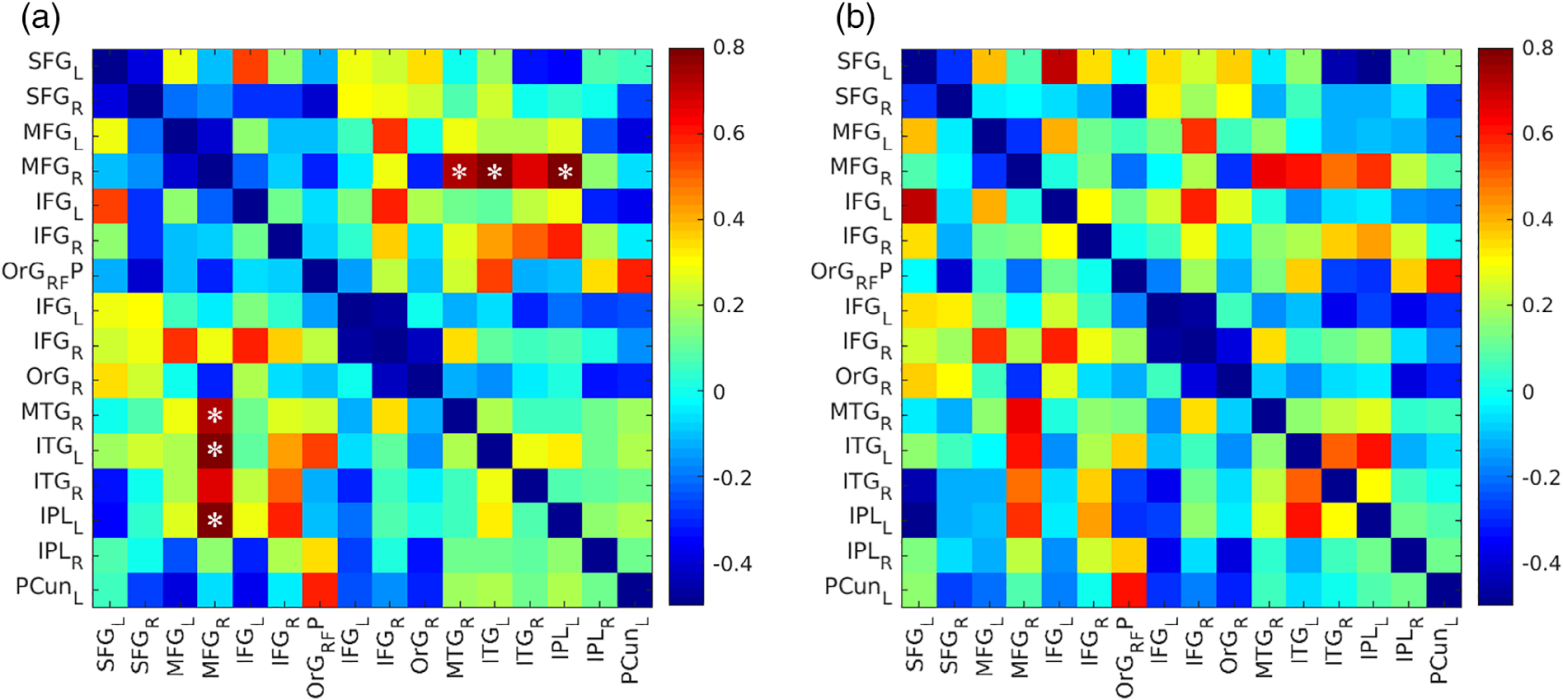
FC differences between FEP subjects and HCs within the FPN and effect sizes. Effect sizes quantified with Cohen's *d*. (a) Differences in FC between the HC and FEP groups before the QPP regression. (b) Differences in FC between the HC and FEP groups after the QPP regression. *p < .05 after FDR correction. FC, functional connectivity; FEP, first‐episode psychosis; FPN, frontoparietal network; HCs, healthy controls; QPP, quasiperiodic pattern

FC matrices were created to quantify the extent of interregional FC between each pair of ROIs within the FPN in both groups. For each functional scan, one FC matrix was created via Pearson's correlation coefficient (*r*) by correlating the mean time courses between each ROI. To compare these correlations in the two groups, the correlation values were Fischer *z*‐transformed (*Z*[*r*]) and arranged into a 16 ROIs × 16 ROIs matrix. The FC matrices from all scans were averaged to obtain the mean FC for each group. Then, FC strength was compared between the HC and FEP groups. To conduct group comparisons, a two‐sample *t*‐test was performed for each ROIs connection to check for a significant change in FC strength. Given that there were 120 (i.e., [16 × 15]/2) connections to compare, multiple comparisons correction was performed using false discovery rate method (FDR) of *p* < .05 (Storey, 2002). All effect sizes were calculated according to the Cohen's *d* (Figure 1a).

### 2.4 Acquisition of QPPs

A spatiotemporal pattern‐finding, correlation‐based iterative algorithm, described by Yousefi et al. (2018) was used to search for repeating patterns in the functional scans. The pattern‐finding algorithm begins by conducting a sliding correlation between a random starting segment within a functional time series and the functional time series itself. If the brain activity captured in the segment repeats at other instances in the functional time series, the resulting sliding correlation vector will contain local maxima (i.e., peaks) indicating those occurrences. At each of those instances, additional segments of the same length are extracted and averaged together into an updated segment. Subsequent sliding correlations are then conducted between the continually updated segment and the functional time series. These steps are repeated until the updated segment no longer shows variation and represents a reliably repeating pattern of brain activity within the functional time series.

**Figure 2:**
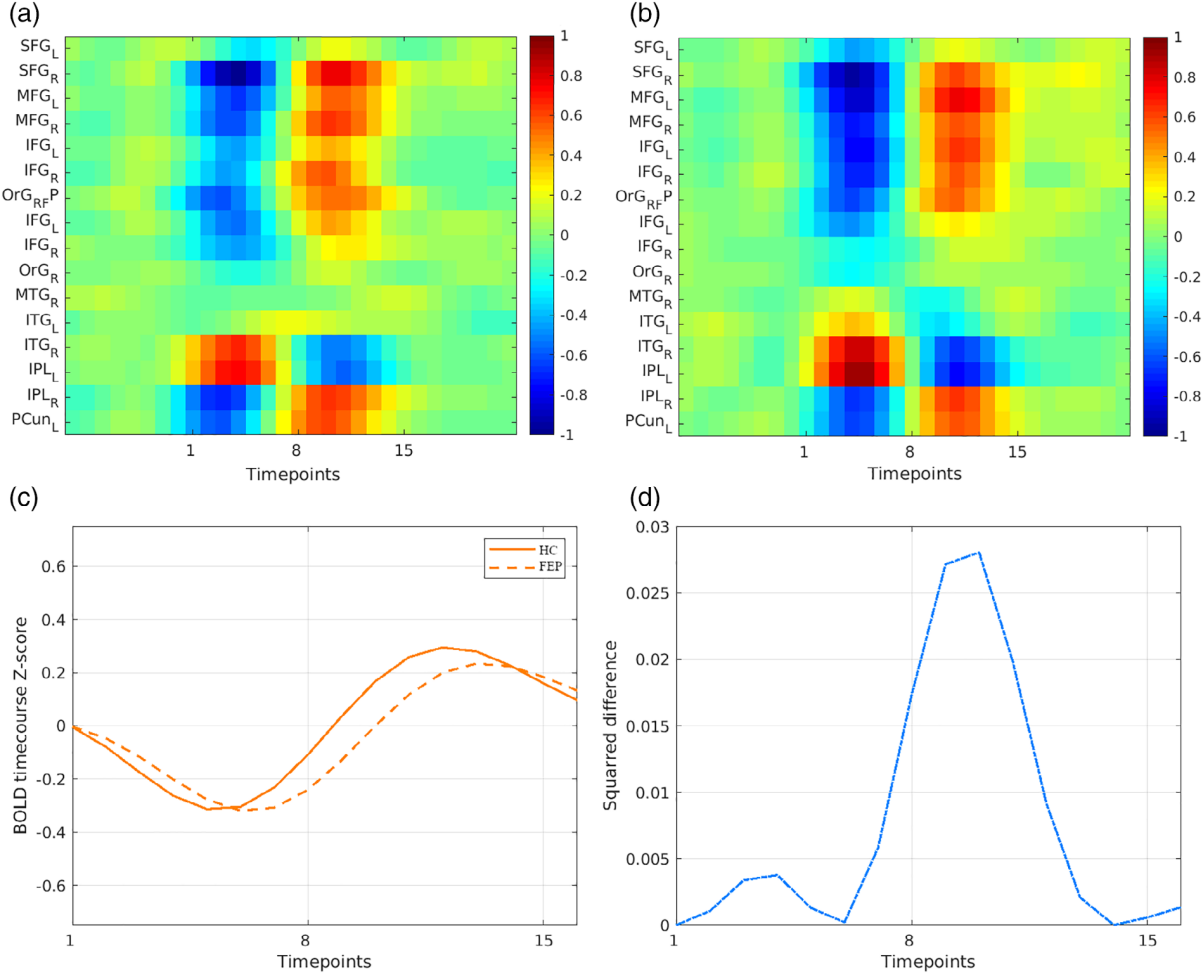
Spatiotemporal comparison of the QPP of HCs and the QPP of FEP subjects. (a) Spatiotemporal pattern of QPP_HC_ and (b) QPP_FEP_ for the 16 ROIs in the FPN. The color bar corresponds to the correlation time course of the QPPs. (c) Mean time course of the QPPs for both groups. (d) The square of the difference between the HC and FEP QPPs time course. 1 time point = 1.55 second. FEP, first‐episode psychosis; HC, healthy controls; QPP, quasiperiodic pattern

QPPs in humans are approximately 20 s long (Majeed et al., 2011); for this study, the window length, or template duration, was set to 15 time points (= 23.2 s). Scans were concatenated for each subject and the QPPs inspected at every time point (Yousefi et al., 2018). Hence, for the template resulting from each time point, values of its sliding correlation at local maxima that were above the threshold of 0.3 at the final iteration were summed and, the template with the highest sum was designated as the most representative QPP for its respective group. Selected in this way, the most representative QPP is guaranteed to have high correlation and large numbers of occurrences relative to other templates. By doing so, one representative QPP was established for the HC group (QPP_HC_), and another representative QPP was established for the FEP group (QPP_FEP_) (Figure 2).

**Figure 3:**
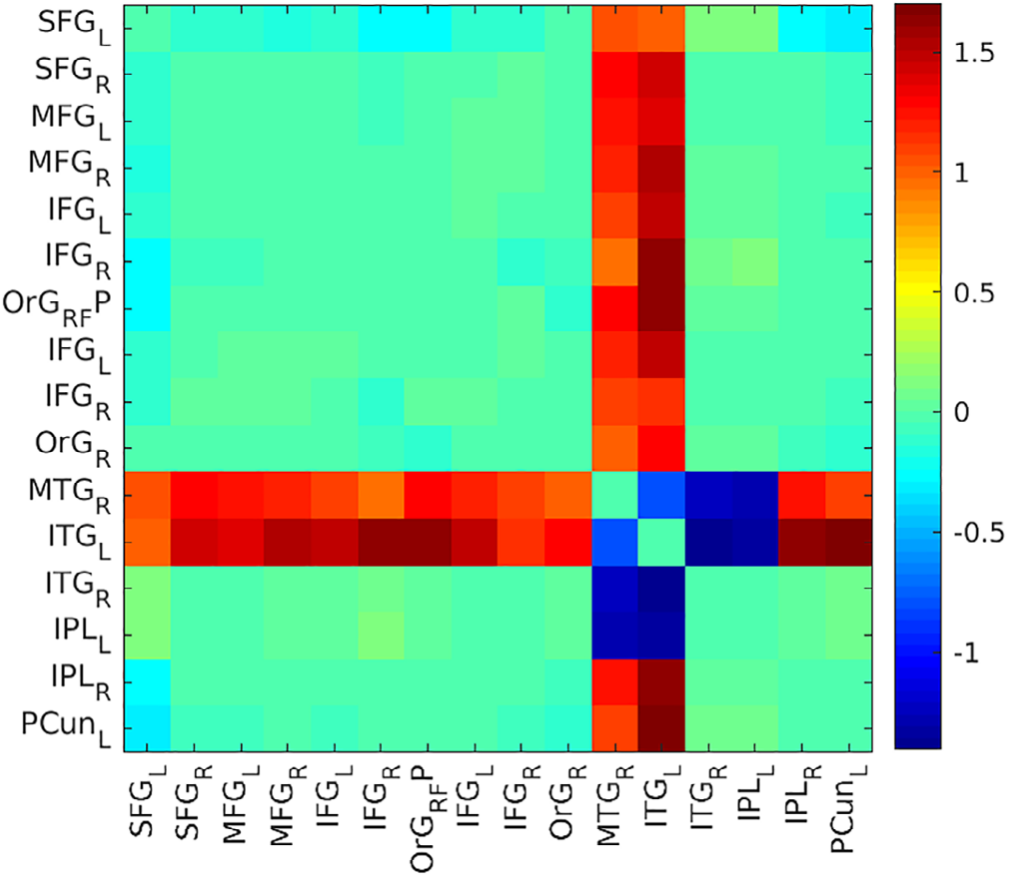
Matrix of group difference of the QPP time course correlation. Differences (*Z*[*r*]) were obtained by a subtraction of the average *z*‐transformed Pearson correlation values of HCs minus FEP patients. The color bar corresponds to a z‐transformed Pearson correlation for the differences. FEP, first‐episode psychosis; HCs, healthy controls; QPP, quasiperiodic pattern

Similar to the analysis of FC data, the QPPs were detected into the 246 ROIs in the Brainnetome ROI atlas, then concatenated according to the Yeo frontoparietal functional network atlas (Yeo et al., 2011) (Figure 3). Then, we also computed Pearson's correlation coefficient (*r*) between each regional QPP time course of one cycle (i.e., a window length of 15 time points) within each groups. And after, to compare regional correlation between the FEP and HC groups, correlation values were *z*‐transformed and then compared using a simple difference (delta value: *Z*[*r*]) (Figure 3).

#### 2.4.1 Basic metrics of the QPPs

The QPPs algorithm has two main outputs: a repeating spatiotemporal pattern from within the functional time series (Figure 2a,b), and a sliding correlation vector of the pattern (Figure S1a,c) within the functional time series itself (Yousefi et al., 2018). The resulting sliding correlation vectors contained local maxima correlation (the peaks), which corresponds to the occurrence of QPPs in the functional scans. Hence, it is possible to characterize basic metrics of the QPPs, like the strength and frequency of the QPPs. The strength of the QPP was defined as the mean height of those peaks. The frequency of the QPP was defined as the rate of occurrence of those peaks over the time course of the resting‐state scans. To compare the strength and frequency of the QPPs across the HC and FEP groups, an arbitrary peak height threshold of 0.3 was chosen. To conduct group comparisons of these basic metrics, a two‐sample *t* test was performed. Moreover, to compare QPP_FEP_ and QPP_HC_, a Pearson correlation test using fine‐phase matching was performed.

#### 2.4.2 Removal of QPPs from functional scans

As QPP involves coactivation of the major networks, it contributes to the FC. Hence FC difference can be compared before and after regression of it (Abbas, Belloy, et al., 2019). Thus, for each functional scan, we created the FC matrix after its QPPs had been regressed out. In the same way that was previously described, we created these FC matrix for both groups (Figure 1b). Specifically, QPPs were removed from the BOLD signal using the regression method described in Abbas, Belloy, et al. (2019). For each functional scan, a unique regressor was calculated for every brain voxel: The sliding correlation of the QPP was convolved with the time course of each brain voxel during the QPP. The obtained regressor was *z*‐scored to match the signal in the functional scan. Next, linear regression was carried out using beta coefficients and the regressors calculated for each brain voxel. By doing so, a functional scan with attenuated presence of the QPP in the BOLD signal was produced. Then, each signal was parceled into the 16 ROIs. Differences in the strength and frequency of QPPs after their removal were compared using the distribution of a sliding correlation vector of the QPPs before and after the QPP regression (Figure S1).

FC strength, before and after regression of QPPs, was compared within and between groups (Figure 1b). Again, multiple comparisons correction was performed using FDR of *p* < .05 (Storey, 2002) and effect sizes were calculated according to Cohen's *d*.

#### 2.4.3 Clinical scale and duration of untreated psychosis

We used the BPRS total score and the RBANS total and attention subscale scores (Petersen & Posner, 2012) to evaluate the relationships between QPPs and symptom severity, as well as QPPs and cognitive performance (Table 1). While all participants were rated with the BPRS the day of the scan, RBANS testing was obtained within days of scanning because of the importance to test subjects in a quiet environment. To assess the relationship between QPP metrics and clinical/cognitive variables, we used Pearson correlations with the significance threshold setup at *p* < .05. In addition, given recent findings of correlations between the duration of untreated psychosis (DUP) (Maximo, Nelson, Armstrong, Kraguljac, & Lahti, 2020), which describes the duration between discernable psychotic symptoms to the time of initial treatment contact and FC, we also explored the relationship between QPPs and DUP. Moreover, we also assessed the relationship between all these indices and the FC.

## 3 RESULTS

### 3.1 Differences in FC before QPPs regression between groups

Significant group differences were found in connectivity in the FPN (Figure 1a). The connectivity between the right MFG (rMFG) and the right middle temporal gyrus (rMTG; *μ* = −0.11 ± 0.25), the left inferior temporal gyrus (lITG; *μ* = 0.12 ± 0.37), and the left inferior parietal (lIPL; *μ* = −0.11 ± 0.28) were weaker in the FEP than HC (*μ* = 0.09 ± 0.27, Cohen's *d* = 0.79, *p* = .038; *μ* = 0.39 ± 0.30, Cohen's *d* = 0.78, *p* = .044; *μ* = 0.09 ± 0.22, Cohen's *d* = .73, *p* = .026, respectively, for rMTG, lITG, and lIPL with rMFG).

### 3.2 Differences in QPPs between groups

#### 3.2.1 Differences in the spatiotemporal pattern

The spatiotemporal pattern QPPs of both groups were quite similar (Figure 2). Although, calculating the square of the difference between time courses in each of the group's QPPs revealed a slight difference (Figure 2d). At the peak where QPP_FEP_ and QPP_HC_ signal were most separated, the mean square difference was 0.028. Moreover, a strong correlation was found between QPP_FEP_ and QPP_HC_ (*r* = .93, *p* < .01). Differences in the basic metrics of the QPP sliding correlations were conducted with all functional scans in each group. In this cumulative sliding correlation, there was a significant group difference in the strength of the QPPs (QPP_HC_: *μ* = 0.42 ± 0.09; QPP_FEP_: *μ* = 0.45 ± 0.10; Cohen's *d* = −0.24, *p* < .01), but not in their frequency (QPP_HC_: *μ* = 8.92 ± 2.01; QPP_FEP_: *μ* = 9.15 ± 2.55; Cohen's *d* = −0.09, *p* = .662).

#### 3.2.2 QPPs correlation matrix

To compare one representative QPP (QPP_HC_) versus another (QPP_FEP_), the mean time course of each ROI was calculated for both QPPs. For each ROI, a Pearson correlation test was conducted between its mean time course in the HC QPP and its mean time course in the FEP QPP. The two main group differences in the QPP time course matrix were found within the rMTG and the lITG (Figure 3). We extracted QPP time course correlations in regions where significant differences in static FC were observed (Figure 1): for FEP the correlation values were rMTG = −.63, lITG = −.89, and for lIPL = −.99 and were numerically stronger for HC (rMTG = .56, lITG = .63, and for lIPL = −.97). Thus delta values of rMTG = 1.19, the lITG = 1.52, and the lIPL = 0.02.

### 3.3 Differences in FC after QPPs regression between groups

FC group differences were compared before and after regression of QPPs. Examples of the sliding correlation vectors and the strength and frequency of the QPPs after QPP regression are shown in Figure S1.

There were no significant group differences in FC after QPPs regression (Figure 1b). In both groups, QPP regression led to an overall decrease in FC in the FPN. Though the overall direction of FC differences was the same, removal of the QPP_FEP_ from FEP scans resulted in far fewer significant changes in FC (with an arbitrary 20% of variance, 82% of between ROI connections within the FPN are modified after QPP regression) compared to removal of the QPP_HC_ from HC scans (always with an arbitrary 20% of variance, 62% of between ROI connections within the FPN are modified after QPP regression).

### 3.4 Clinical scale and duration of untreated psychosis

#### 3.4.1 Relationships with symptoms

In FEP subjects, we did not find correlations between BPRS and DUP (BPRS total, *r* = −.12, *p* = .44). Moreover, for FEP subjects, we did not find correlations between QPP_FEP_ and BPRS, or between QPP_FEP_ and DUP regarding the basic metrics of strength (DUP, *r* = −.27, *p* = .09; BPRS, *r* = −.05, *p* = .75) and frequency (DUP, *r* = −.04, *p* = .77; BPRS, *r* = −.05, *p* = .76) (Table 2).

**Table 2:** Correlations between BPRS or RBANS and QPP basic metrics

	HC	FEP
	*n* = 40	*n* = 40
BPRS total		
QPP strength	–	*r* = −.05, *p* = .75
QPP frequency	–	*r* = −0.05, *p* = .76
RBANS total		
QPP strength	*r* = −.22, *p* = .22	*r* = .11, *p* = .52
QPP frequency	*r* = −.20, *p* = .25	*r* = .09, *p* = .57
RBANS attention subscale		
QPP strength	*r* = −.05, *p* = .75	*r* = .15, *p* = .35
QPP frequency	*r* = −.24, *p* = .18	*r* = −.15, *p* = .36

Abbreviations: BPRS, brief psychiatric rating scale; FEP, first‐episode psychosis; HCs, healthy controls; QPP, quasiperiodic pattern; RBANS, repeatable battery for the assessment of neuropsychological status.

We did not find any significant relationships between BPRS or DUP and the FC in the FPN ROIs (see Figures S2 and S3).

#### 3.4.2 Relationships with cognitive function

We did not find correlations between the RBANS attentional subscale (or the RBANS total) and the basic metrics of the QPP in neither strength (RBANS attentional subscale: QPP_FEP_, *r* = .15, *p* = .35; QPP_HC_, *r* = −.05, *p* = .75) nor frequency of the QPP (RBANS attentional subscale: QPP_FEP_, *r* = −.15, *p* = .36; QPP_HC_, *r* = −.24, *p* = .18) in FEP and HC subjects (Table 2).

We did not find any significant relationships between RBANS total/attentional and the FC in the FPN ROIs (see Figure S4) in FEP and HC subjects.

## 4 DISCUSSION

The aim of the current study was to examine whether altered FC within the FPN before antipsychotic drug administration constitutes a potential trait alteration in antipsychotic‐naïve FEP. In line with our hypothesis, we found a pattern of disrupted connectivity within this network in FEP. In addition, we found that the strength of the correlation of the QPP in the QPP sliding vector was significantly stronger in FEP than in HC, suggesting a greater impact of the QPP to intrinsic brain activity in these subjects. Finally, in FEP, regressing QPP from the functional scans erased all significant group difference in FC, suggesting that abnormal FC in FEP could result from altered QPP.

### 4.1 FPN connectivity

The FPN is the portion of the control system involved in highly adaptive processes of goal‐directed task demands (Cole et al., 2013). If disrupted, it could predispose to some of the symptoms of mental illness (Schmidt et al., 2015), especially through dysregulation of feedback control mechanisms via impaired dynamic connectivity (Cole et al., 2014).

Our findings of hypoconnectivity within the FPN in antipsychotice‐naïve FEP are in agreement with the results of others (Baker et al., 2014, 2019; Ren et al., 2013), but is in contrast to other studies focused on functional networks in antipsychotic‐naïve FEP showing patterns of hyperconnectivity (Anhøj et al., 2018; Li et al., 2015). This discrepancy could be related to their use of an ICA method across the whole brain instead of just the FPN. Moreover, the difference can be due to global signal regression not being done in these studies, it is possible that global signal may modulate the associations between resting‐state FC and behavior (Li et al., 2019). In any case, these results suggest that the observed alterations are not a function of antipsychotic medication exposure or illness chronicity, and therefore may be regarded as trait specific.

We observed significant FPN hypoconnectivity between the MFG (frontal lobe) and the middle and inferior temporal gyri (temporal lobe), as well as the inferior parietal gyrus (parietal lobe). These key goal‐directed planning regions are important for information processing across brain networks (Buckner, Andrews‐Hanna, & Schacter, 2008). In particular, the right MFG may play an important role in reorienting attention from exogenous to endogenous attentional control (Japee, Holiday, Satyshur, Mukai, & Ungerleider, 2015), the MTG and the ITG have been linked to multimodal sensory integration (Onitsuka et al., 2004), and the IFG as a core system for goal‐directed task sets (Dosenbach et al., 2006). An alteration of the connectivity among these regions could support the core symptoms of psychotic illness, like thought distortions and anomalous self‐experience (Buckner et al., 2008; Northoff, 2015).

### 4.2 Implication of QPPs for FEP

Few studies have characterized aberrant dynamic network in early illness SZ (Du et al., 2018; Lottman et al., 2017) and in subjects experiencing psychotic‐like experiences (Barber, Lindquist, DeRosse, & Karlsgodt, 2018). However, the contribution of QPPs to FC has never been investigated in the psychosis spectrum before.

QPPs were observed in both FEP and HC. Spatiotemporal patterns in FPN, between FEP and HC were largely similar, as indicated by the strong correlation between the two QPPs. Although the frequency of the spatiotemporal pattern was not different between the groups, a key group difference was found in the strength of the QPP correlation via the QPP sliding vector. The correlation of the QPP with all functional scans was significantly stronger for FEP compared to HC, suggesting a greater impact of the QPPs to intrinsic brain activity in the FPN among FEP.

Interestingly, visual inspection of group difference for the correlation of QPPs time course between regions reveals abnormal patterns in the rMTG and the lITG (Figure 3), regions where we also saw aberrant static FC patterns. Because it has been previously shown that QPPs contribute to FC in the brain (Abbas, Belloy, et al., 2019), we also regressed QPPs from their functional scans, which erased all significant group difference in FC. In addition, after QPP regression, we observed that a greater number of connections were affected by this regression in FEP than in HC. Taken together, these findings support the proposition that FC group differences in the FPN among FEP could result from altered strength of the QPP.

In SZ, excess levels of dopamine release have been consistently reported (Lieberman & First, 2018; Lieberman, Kinon, & Loebel, 1990). Interestingly, there is some evidence that dopamine modulation is important for the generation of infra‐slow oscillations (Kobayashi, Shimada, Fujiwara, & Ikeguchi, 2017) which are associated with QPPs (Thompson et al., 2014). Speculatively, in the early phase of psychosis, increased dopamine could lead to abnormal infraslow oscillations, via abnormal regulation of *N*‐methyl‐d‐aspartate receptor in the FPN (Schmidt et al., 2015), followed by QPP and FC alterations. In addition, the low‐frequency fluctuations in the BOLD signal, which is also linked to infraslow electrical activity of the electroencephalographic signal (Grooms et al., 2017; Hiltunen et al., 2014), has been reported as altered in psychosis and early illness SZ (Fryer et al., 2016; Meda et al., 2015; Ren et al., 2013).

### 4.3 Clinical assessment, DUP, and FPN connectivity

Neither static FC or QPP metrics were related to symptom severity (assessed using the BPRS), which is in line with previous findings (Baker et al., 2014). Likewise, we did not find the DUP to be associated with either static FC or QPP metrics. This is surprising considering recent findings of association between the FC of several known networks and the DUP (Manivannan et al., 2019; Maximo et al., 2020; Sarpal et al., 2017).

Moreover, there was no association between static FC or QPP metrics and cognitive function, as measured with the RBANS. Integration across more distributed regions than the FPN may be needed to support attentional and global functions (alerting, orienting, and executive control) (Berger & Posner, 2000; Mackie, Van Dam, & Fan, 2013).

### 4.4 Limitations

As addressed by Abbas, Belloy, et al. (2019), the method used to regress the QPPs from the BOLD time course inherently assumes that QPPs are an additive component to the remaining BOLD signal. The assumption is based on multimodal experiments in rodents (Thompson et al., 2014) and need to be further explored in humans.

## 5 CONCLUSIONS

We report disruption of the FPN in antipsychotic‐naïve FEP relative to HCs, supporting the proposition that this dysfunction is trait specific. In addition, we found abnormal QPP metric in FEP, suggesting a greater impact of the QPP to intrinsic brain activity. A better understanding of the contribution of the QPPs to FC in FEP may shed light into the mechanisms of functional dysconnectivity in mental illness.

## CONFLICT OF INTEREST

The authors declare no potential conflict of interest.

## Supporting information


**Figure S1** Examples of sliding correlation vector acquired with 2 randomly selected concatenated functional scans before (blue) and after (red) the quasiperiodic pattern regression for A) Healthy controls (HC) and C) First‐episode psychosis patients (FEP). The resulting sliding correlation vectors contained local maxima correlation, which corresponds to high probability to have a QPP in the functional scans. B) Distribution of correlation values between the most representative QPP and the sliding correlation vectors, before (blue) and after (red) QPP regression, for HC and for D) FEP. The ordinate ax of the histogram give the frequency of these correlation values (i.e., the occurrence of QPPs in the functional scans) and by averaging the abscissa frequency cells, it is possible to obtain the strength of the sliding correlation vectors. 1 time points = 1.55 second.
**Figure S2**. Correlation of the brief psychiatric rating scale (BPRS) and functional connectivity in the 16 ROIs within the frontoparietal network (FPN). Lower triangle: P‐value map before FDR correction. Upper triangle: P‐value map after FDR correction. The color bar corresponds to p‐value intensities.
**Figure S3**. Correlation of the duration of untreated psychosis and functional connectivity in the 16 ROIs within the frontoparietal network. Lower triangle: P‐value map before FDR correction. Upper triangle: P‐value map after FDR correction. The color bar corresponds to p‐value intensities.
**Figure S4**. Correlation of the repeatable battery for the assessment of neuropsychological status (RBANS) and functional connectivity in the 16 ROIs within the frontoparietal network. A) Correlation with RBANS total in Healthy controls (HC) B) Correlation with RBANS total in First‐episode psychosis patients (FEP) C) Correlation with RBANS Attention subscale in HC D) Correlation with RBANS Attention subscale in FEP. In each case, lower triangle: P‐value map before FDR correction. Upper triangle: P‐value map after FDR correction. The color bar corresponds to p‐value intensities.

## Data Availability

MRI data might be obtained upon request by contacting the corresponding author.
