# Supplementary material for: Aberrant static and dynamic functional patterns of frontoparietal control network in antipsychotic‐naïve first‐episode psychosis subjects
Source: Hum Brain Mapp. 2020 May 6;41(11):2999–3008. doi: 10.1002/hbm.24992 (PMC7336157; doi:10.1002/hbm.24992)
Supplement: Supplementary file 1 — Figure S1 Examples of sliding correlation vector acquired with 2 randomly selected concatenated functional scans before (blue) and after (red) the quasiperiodic pattern regression for A) Healthy controls (HC) and C) First‐episode psychosis patients (FEP). The resulting sliding correlation vectors contained local maxima correlation, which corresponds to high probability to have a QPP in the functional scans. B) Distribution of correlation values between the most representative QPP and the sliding correlation vectors, before (blue) and after (red) QPP regression, for HC and for D) FEP. The ordinate ax of the histogram give the frequency of these correlation values (i.e., the occurrence of QPPs in the functional scans) and by averaging the abscissa frequency cells, it is possible to obtain the strength of the sliding correlation vectors. 1 time points = 1.55 second. Figure S2. Correlation of the brief psychiatric rating scale (BPRS) and functional connectivity in the 16 ROIs within the frontoparietal network (FPN). Lower triangle: P‐value map before FDR correction. Upper triangle: P‐value map after FDR correction. The color bar corresponds to p‐value intensities. Figure S3. Correlation of the duration of untreated psychosis and functional connectivity in the 16 ROIs within the frontoparietal network. Lower triangle: P‐value map before FDR correction. Upper triangle: P‐value map after FDR correction. The color bar corresponds to p‐value intensities. Figure S4. Correlation of the repeatable battery for the assessment of neuropsychological status (RBANS) and functional connectivity in the 16 ROIs within the frontoparietal network. A) Correlation with RBANS total in Healthy controls (HC) B) Correlation with RBANS total in First‐episode psychosis patients (FEP) C) Correlation with RBANS Attention subscale in HC D) Correlation with RBANS Attention subscale in FEP. In each case, lower triangle: P‐value map before FDR correction. Upper triangle: P‐value map after FDR correctio [file HBM-41-2999-s001.docx]

# Supplementary data


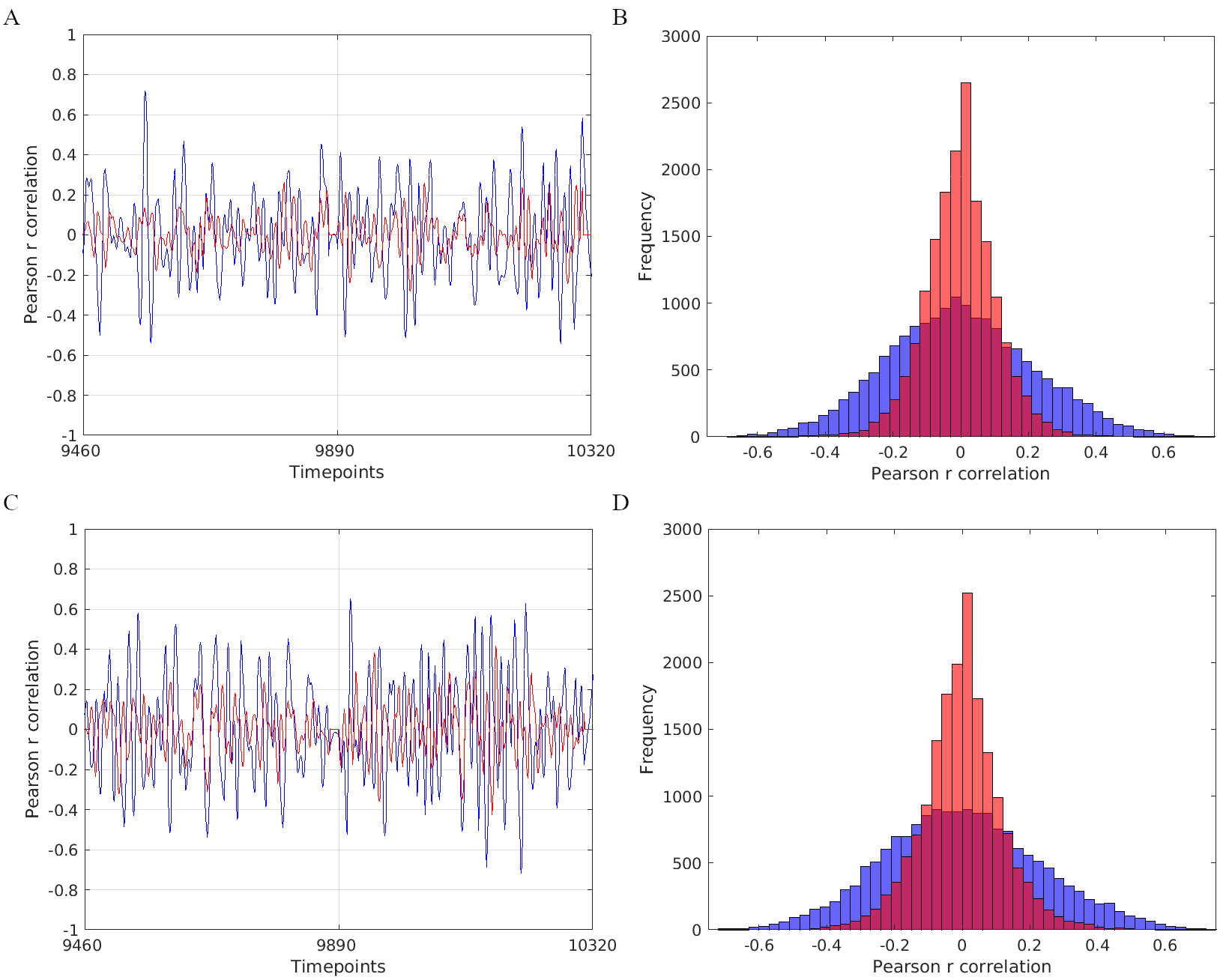


***Fig. 1 Supplementary.*** *Examples of sliding correlation vector acquired with 2 randomly selected concatenated functional scans before (blue) and after (red) the quasi-periodic pattern regression for* ***A****) Healthy controls (HC) and* ***C****) First episode psychosis patients (FEP).* ***The resulting sliding correlation vectors contained local maxima correlation, which corresponds to high probability to have a QPP in the functional scans.* *B) Distribution of correlation values between the most representative QPP and the sliding correlation vectors, before (blue) and after (red) QPP regression, for HC and for D) FEP. The ordinate axe of the histogram give the frequency of these correlation values (i.e. the occurrence of QPPs in the functional scans) and by averaging the abscissa frequency cells, it is possible to obtain the strength of the sliding correlation vectors. 1 timepoints=1.55 second.***


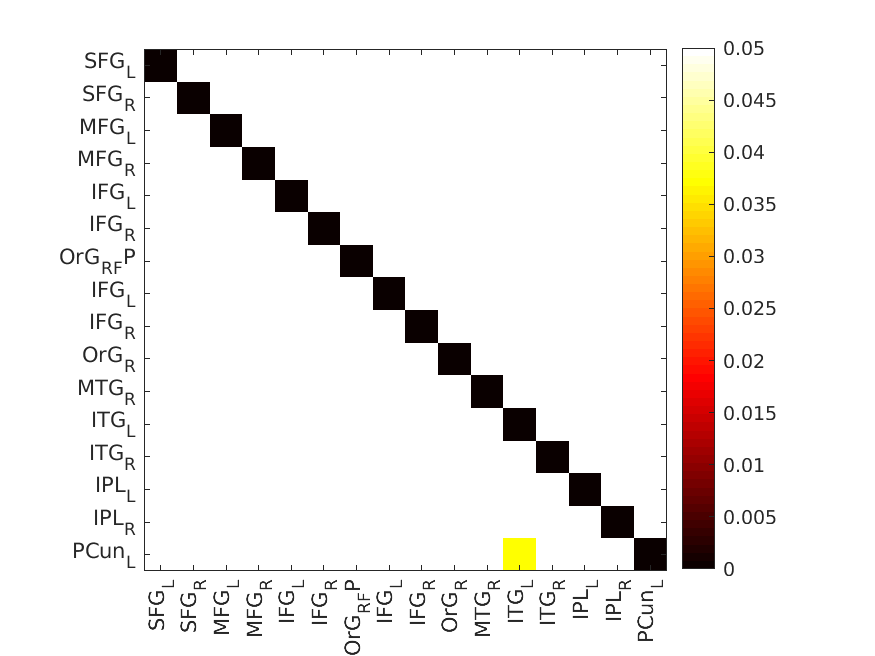


***Fig. 2 Supplementary.*** *Correlation of the brief psychiatric rating scale (BPRS) and functional connectivity in the 16 ROIs within the fronto-parietal network (FPN). Lower triangle: P-value map before FDR correction. Upper triangle: P-value map after FDR correction. The color bar corresponds to p-value intensities.*


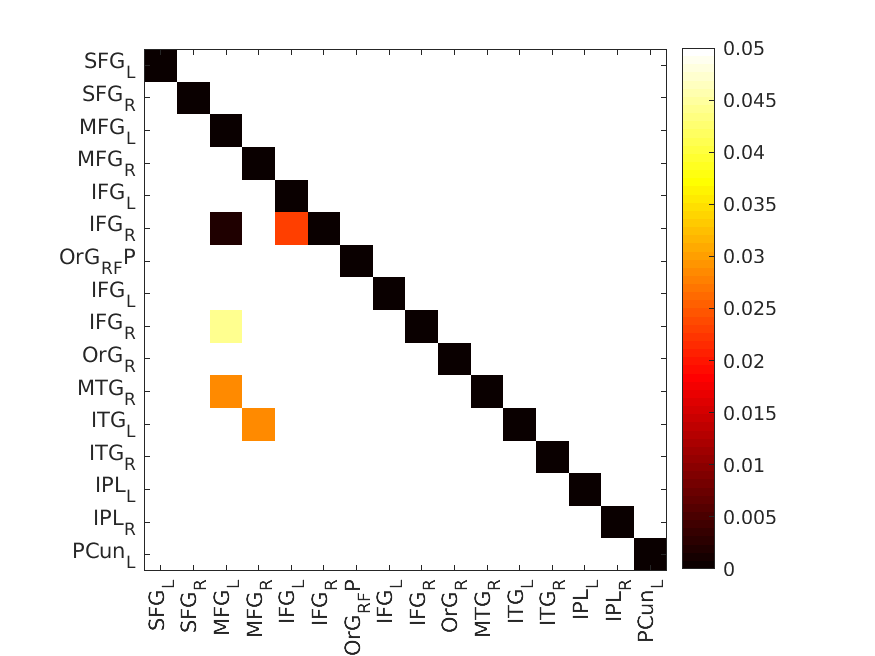


***Fig. 3 Supplementary.*** *Correlation of the duration of untreated psychosis and functional connectivity in the 16 ROIs within the fronto-parietal network. Lower triangle: P-value map before FDR correction. Upper triangle: P-value map after FDR correction. The color bar corresponds to p-value intensities.*


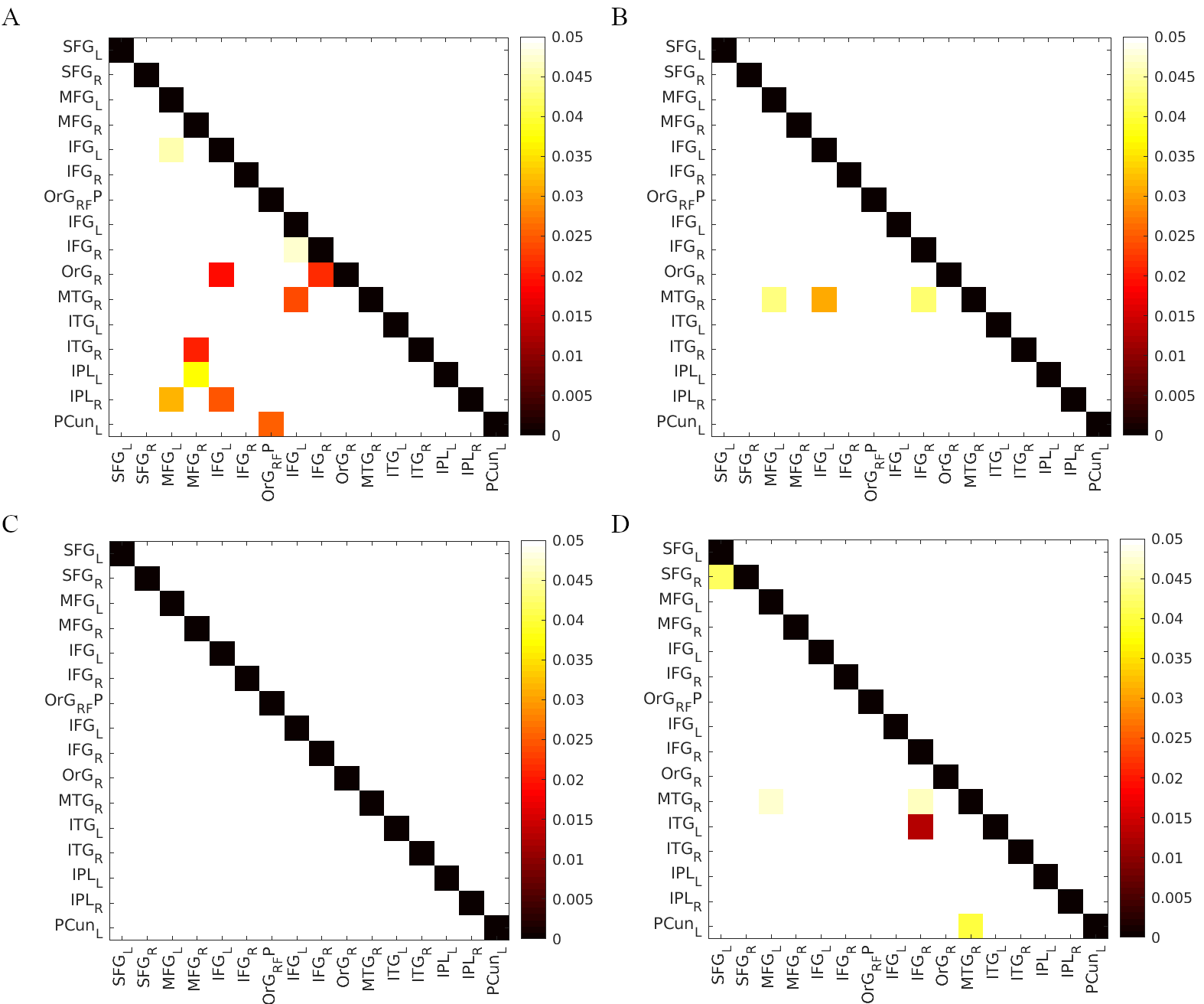


***Fig. 4 Supplementary.*** *Correlation of the repeatable battery for the assessment of neuropsychological status (RBANS) and functional connectivity in the 16 ROIs within the fronto-parietal network.* ***A****) Correlation with RBANS total in Healthy controls (HC)* ***B)*** *Correlation with RBANS total in First episode psychosis patients (FEP)* ***C****) Correlation with RBANS Attention subscale in HC* ***D)*** *Correlation with RBANS Attention subscale in FEP. In each case, lower triangle: P-value map before FDR correction. Upper triangle: P-value map after FDR correction. The color bar corresponds to p-value intensities.*
